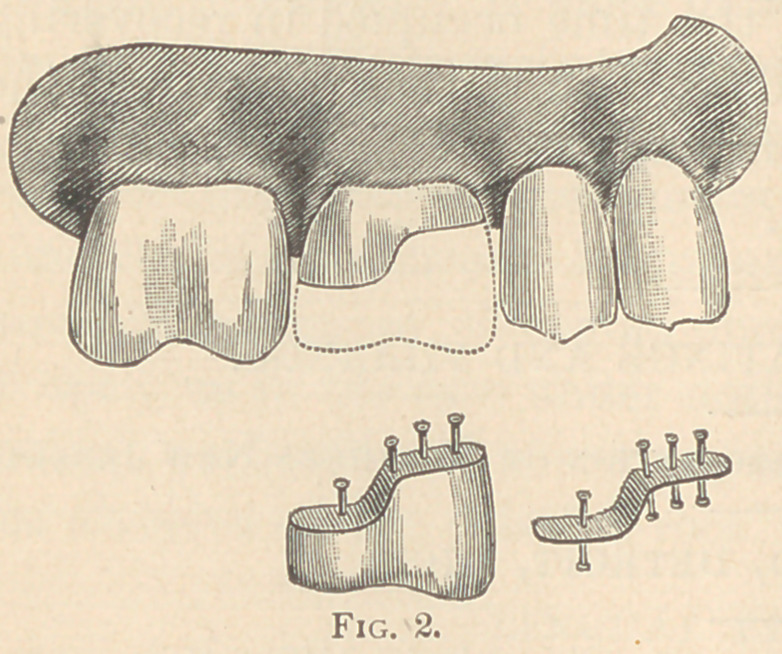# Metallic Enamel Coatings and Fillings

**Published:** 1887-08

**Authors:** C. H. Land

**Affiliations:** Detroit, Mich.


### METALLIC ENAMEL COATINGS AND FILLINGS.

READ BEFORE THE CENTRAL DENTAL ASSOCIATION OF NORTHERN NEW JERSEY.

BY DR. C. H. LAND, DETROIT, MICH.

In the absence of practical demonstrations it is difficult to comprehend all the advantages brought about by improvements. The accompanying engravings, Figs. 1 and 2, are taken from practical cases that have at this date been in use for one year. In the case represented by Fig. 1, the patient was about sixty years of age.

FIG. 1.

The right lateral incisor was prepared with a Howe post, shown in its relative position. The five remaining teeth, after the cavities were prepared, contained tooth substance as represented by the dark surfaces, the white representing the lost portion of each tooth, restored with sections of porcelain made to imitate the exact color and contour of the original tooth substance. The cavities are prepared as for gold filling, when a thin piece of annealed platinum plate, No. 35 standard gauge, is placed over the tooth, and by means of burnishers made to take a perfect impression of the outer rim of the cavity, after which platinum pins are attached, as shown at A. The object of the pins is to serve as a fastening, both for the porcelain paste or body and as retainers to hold the completed section in the cavity of the tooth. The porcelain paste or body is built upon the platinum disk and made to imitate the lost portion of the tooth. It is then baked in a gas furnace, requiring but twenty minutes for the first biscuit and fifteen for the second, and when finished it appears as shown at B, ready to be cemented with oxy-phosphate. C and D are modifications for the other teeth, and Fig. 2 illustrates porcelain facings for molars.

Fig. 2.

The especial feature of this system, to which I wish to call your attention, is the large amount of tooth substance preserved above the gum, there being no necessity for telescoping the root so far below as to sever the tissues. This mode of practice also dispenses with the long operations and protracted use of the rubber dam; it almost entirely obviates the use of amalgam, and saves the necessity for large gold fillings; there is no malleting, no long and tedious operation either for the patient or dentist, while at the same time teeth are perfectly restored, both in appearance and usefulness.

There is another advantage in the use of the enamel coatings which is not, in my opinion, a trivial matter. When large metallic fillings are inserted, the constant thermal changes consequent upon their alternate heating and cooling must exercise an unfavorable influence upon the tissues about the tooth. Even if the pulp be dead and the root be filled, there will be a checking and fracture of the tooth in time, from the continually varying changes of temperature. An inflammation of the membranes will also be likely to occur from the same cause, and thus the tooth will in time be lost from the mere influence of the presence of a large mass of metal.

It is also a fact that large gold fillings cannot be inserted without so much malleting that the strength of the tooth is gone, and frail walls are cracked beyond the possibility of repair. These dangers are all obviated by the use of the porcelain facings, while teeth so restored are much more natural in feeling and more grateful to the touch of the tongue than any metallic filling can be.

## Figures and Tables

**Fig. 1. f1:**
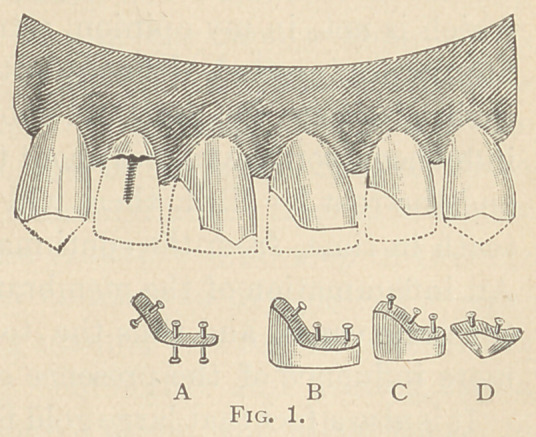


**Fig. 2. f2:**